# Enhancing groundwater level prediction with a hybrid deep learning model in Jinan City, China

**DOI:** 10.1038/s41598-025-28200-5

**Published:** 2025-12-24

**Authors:** Can Zhuang, Liangliang Cui, Yi Cui

**Affiliations:** 1 School of Civil Engineering and Geomatics, Shandong University of Technology, Zibo, China; 2 Jinan Zhongan Digital Technology Co., Ltd, Jinan, China; 3 Jinan Qilu Shutong Technology Co., Ltd, Jinan, China

**Keywords:** GWL prediction, Deep learning, Spatio-temporal features, Jinan city, Engineering, Environmental sciences, Hydrology, Mathematics and computing

## Abstract

Accurate prediction of groundwater levels (GWL) is critical for sustainable utilization and scientific management of groundwater resources. However, precise forecasting of GWL fluctuations faces significant challenges due to the complex nonlinear coupling effects of hydrogeological conditions and hydro-meteorological factors. In recent years, research on GWL prediction based on deep learning models has become a cutting-edge topic in the field of hydrogeology. This study focused on Jinan City, China, and constructed a novel hybrid deep learning model that integrates graph neural networks to capture spatial relationships and recurrent neural networks to model temporal dynamics, effectively learning the complex spatio-temporal patterns in the data, namely the Spatio-Temporal Graph Prediction Model (STGPM). Our approach uniquely captures both hydrological connectivity between monitoring wells and multi-scale temporal dependencies, overcoming key limitations of conventional time-series models. Comparative experiments demonstrate that STGPM outperforms the benchmark models on the test set, achieving the lowest prediction errors (MAE = 0.039, RMSE = 0.052) and the highest coefficient of determination (R^2^=0.988). Notably, for the monitoring well data not involved in model training, the STGPM still maintains excellent predictive accuracy (MAE = 0.062, RMSE = 0.087, R^2^=0.980), demonstrating the model’s strong generalization ability to unmonitored locations. This study provides water resource managers with a reliable decision-support tool for sustainable groundwater management and spring conservation strategies. The proposed methodological framework also offers a transferable solution for addressing various environmental forecasting challenges characterized by spatial heterogeneity.

## Introduction

Groundwater resources, as the most abundant and valuable freshwater resources globally, play a crucial role in various key areas vital to human activities, such as agricultural irrigation, industrial production, and potable water supply^1–3^. However, global groundwater systems are currently facing multiple pressures, including overexploitation, environmental pollution, and climate change, which have led to a marked deterioration in both the quantity and quality of groundwater^4^. A representative case is Jinan City, China, renowned as the “Spring City” for its iconic karst spring system, with four major spring groups—Baotu Spring, Heihu Spring, Pearl Spring, and Wulongtan Spring—distributed within its territory, and it has a rich variety of groundwater types^5^. With the rapid development of socio-economy and the continuous advancement of urbanization, the area of spring recharge zones is sharply decreasing. The significant increase in surface imperviousness has severely impaired the infiltration and recharge capacity of karst water, disrupting the natural balance of the regional groundwater system. In recent years, the decline in groundwater levels in the spring distribution areas has posed a severe threat to sustainable spring outflow. Groundwater level (GWL) is a key indicator for measuring the availability and accessibility of groundwater and is closely related to various hydrological and ecological processes^6–8^. Consequently, accurate GWL prediction constitutes not only an essential foundation for groundwater conservation and ecosystem protection but also a prerequisite for formulating sustainable water management strategies and realizing sustainable utilization^9,10^.

However, GWL prediction constitutes a complex systemic process, where dynamic variations are a comprehensive response to coupled interactions of climatic, topographic, and hydrogeological factors^11,12^. This inherent complexity poses significant challenges for precise GWL modeling. Recent advances have witnessed global efforts in developing quantitative and qualitative prediction approaches to establish high-accuracy, robust GWL forecasting models. Current methodologies for groundwater simulation primarily follow two paradigms: physically-based numerical models and data-driven artificial intelligence models. Physically-based numerical models, such as MODFLOW^13^ and FEFLOW^14^, simulate groundwater flow by solving governing partial differential equations derived from physical laws using numerical discretization techniques (e.g., finite difference^15^, finite element^16^, and finite volume methods^17^. These models offer a notable advantage by explicitly elucidating the physical processes driving GWL fluctuations^18^. Nevertheless, the prediction accuracy of such methods is inherently constrained by two critical limitations: the difficulty in accurately parameterizing complex surface water potential fields and the frequent unavailability of precise hydrogeological parameters^10,19^. Furthermore, their high demands for computational resources and data volume often hinder the precise scenario simulation and real-time forecasting^20,21^. These limitations have sparked growing interest among researchers in data-driven artificial intelligence approaches. Benefiting from their strong capability for nonlinear pattern recognition, artificial intelligence methods have demonstrated remarkable advantages and application potential in groundwater forecasting, effectively overcoming the constraints of traditional statistical techniques^22^.

Machine learning (ML), a vital research domain within artificial intelligence, uncovers complex mappings between predictors and response variables from historical data by eliminating the need for explicit representation of physical characteristics or underlying mechanisms, thereby providing a viable alternative to computationally intensive physical models^23^. Numerous studies have successfully integrated meteorological data with GWL datasets to train ML models^24,25^, including support vector machines^26,27^, random forests^28^, and artificial neural networks^29^. However, these individual ML models often struggle to address prediction uncertainties arising from model parameterization and structural limitations^23^. To address these challenges, hybrid ML models have emerged as valuable tools in groundwater simulation. By combining the predictive capabilities of multiple ML algorithms, the hypothesis space for groundwater dynamics prediction can be effectively expanded, thereby enabling more comprehensive analysis of complex factor interactions^4^. For instance, Pham, et al. ^30^ conducted an in-depth investigation into the performance of seven ML models for GWL prediction, demonstrating that the ensemble learning methods Bagging-RT and Bagging-RF outperformed the other five ML models. Despite the advancements represented by these ML and hybrid models, they primarily remain limited to point-based forecasting, failing to incorporate the spatial interdependencies between monitoring locations, a critical factor in aquifer systems. Furthermore, their performance is often hampered by sensitivity to hyperparameter selection and feature engineering^31^.

To enhance the robustness and accuracy of ML models, researchers integrated them with meta-heuristic optimization algorithms (e.g., Particle Swarm Optimization, Genetic Algorithm) for automated hyperparameter tuning^9,32–34^. For instance, Saroughi, et al. ^35^ employed the Honey Badger Algorithm (HBA) to optimize parameters of ANN and SVR models, with systematic evaluations confirming that the optimized HBA-ANN and HBA-SVR models significantly outperformed their standalone counterparts. In further research, the team integrated ANN with both Coot and Honey Badger optimization algorithms for GWL prediction in the Tabriz plain of Iran^36^. Statistical metric selection based on the Shannon entropy criterion verified the superior predictive performance of the Honey Badger optimization algorithm. This hybridization, as evidenced by studies like Thakur and Karmakar^37^, led to noticeable performance improvements. Nevertheless, while these optimized hybrids addressed parameterization issues, their ability to learn and generalize from the complex, coupled spatio-temporal dynamics inherent in groundwater systems remained inadequate. In addition, although the intelligent optimization algorithms mentioned above demonstrate advantages in efficiency, the present study—considering the small number and discrete nature of the model’s hyperparameters—employs the more comprehensive and stable Grid Search method to ensure the optimality and reproducibility of the results.

Deep learning, a significant branch of machine learning, leverages deep neural architectures with high-parameter capacity to effectively capture high-order nonlinear features and complex correlation patterns in data. Substantial empirical research has demonstrated the superior predictive performance of deep learning approaches over both standalone and hybrid ML methods in water resources management tasks^12,20,38^. Models such as Long Short-Term Memory (LSTM) networks and Gated Recurrent Units (GRU) have become the benchmark for time-series forecasting in hydrology^39,40^. More recently, architectures like the Transformer have been explored for their superior ability to capture long-range dependencies through self-attention mechanisms^41^. Concurrently, Graph Neural Networks (GNNs), such as GraphSAGE^42^, have emerged as powerful tools for modeling relational data and spatial correlations, showing great potential in applications like water quality prediction. A nascent body of research has begun to explore the integration of these temporal and spatial architectures for spatio-temporal forecasting. Studies like Chen et al. ^43^ have proposed hybrid models (e.g., STGCN) combining graph convolutional networks with temporal modules, demonstrating promising results for regional-scale GWL prediction. Despite these advancements, a significant research gap persists. Inherently, GWL prediction is a quintessential complex spatio-temporal problem^43^. From a temporal perspective, GWL dynamics exhibit pronounced periodic fluctuations driven by meteorological conditions and seasonal cycles. Spatially, the inherent circulation mechanisms of groundwater cause water level changes in geographically adjacent areas to display strong spatial correlation. Existing research primarily leverages the nonlinear fitting capabilities of machine learning or deep learning to construct predictive models based on the autocorrelation (which measures the correlation of a time series with its own lagged values) and periodic characteristics of time-series data. However, these methods, relying solely on the autocorrelation features of time series, struggle to effectively characterize the spatial heterogeneity among monitoring wells, thereby limiting the prediction accuracy of the models.

To bridge this gap, this study developed a hybrid deep learning model that integrates spatiotemporal features, aiming to provide a scientific decision-support tool for groundwater management in Jinan City. The primary objectives and innovations of this work were threefold:

1. We developed a new deep learning framework that synergistically integrates GraphSAGE and a multi-branch GRU network, which successfully captured both the hydrological connectivity between monitoring wells and multi-scale temporal dynamics of groundwater systems. This design allowed the model to jointly capture immediate responses to rainfall events, seasonal fluctuations, and inter-annual trends;
2. We introduced a trainable cross-attention mechanism to dynamically fuse the multi-scale temporal features with the spatially-aware graph embeddings, replacing simple concatenation or averaging. This enabled more effective and context-aware integration of spatio-temporal information.
3. We designed a dedicated unseen-well test set to rigorously evaluate the model’s spatial extrapolation ability. The superior performance on this test set demonstrates that our model learns universal hydrogeological patterns rather than merely memorizing site-specific sequences.

The remainder of this paper is organized as follows: Section “Materials and methods” describes the study area, data sources, preprocessing procedures, and the detailed architecture of the proposed STGPM model. Section “Results and discussion” presents the experimental results, including model performance comparisons, ablation studies, and interpretability analysis. Finally, section “Conclusion” concludes the study.

## Materials and methods

**Fig. 1:**
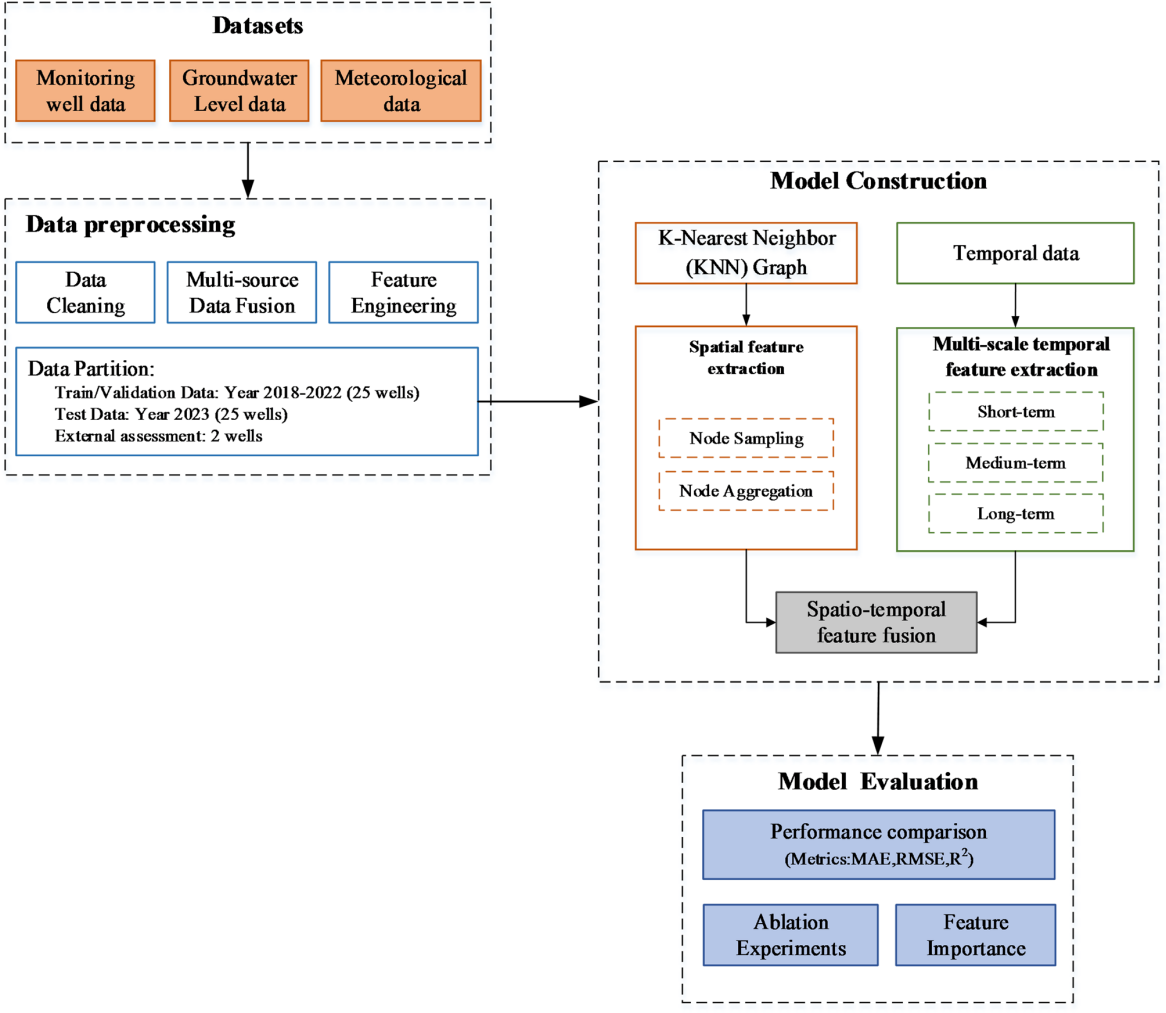
Workflow diagram of the methodology.

The complete workflow of the methodology in this study was divided into three critical stages: data preprocessing, model construction, and model optimization and evaluation (Fig. 1). During the data preprocessing stage, a variety of techniques, including data cleaning and feature engineering, were employed to reconstruct raw data to meet the requirements for model training. The data were also reasonably partitioned to ensure their quality and usability. The modeling phase developed an architecture specifically designed to capture both the temporal dependencies and spatial correlations inherent in GWL dynamics, establishing a robust foundation for prediction tasks. During the model optimization and validation stage, hyperparameters were iteratively optimized using a loss function and an optimizer until convergence, resulting in the optimal model performance. A comprehensive performance validation was conducted through a multi-indicator, multi-dimensional evaluation system. The experimental design included performance comparison, ablation experiments, and model interpretability analysis to ensure the accuracy and reliability of the model.

### Study area and datasets

#### Study area

**Fig. 2:**
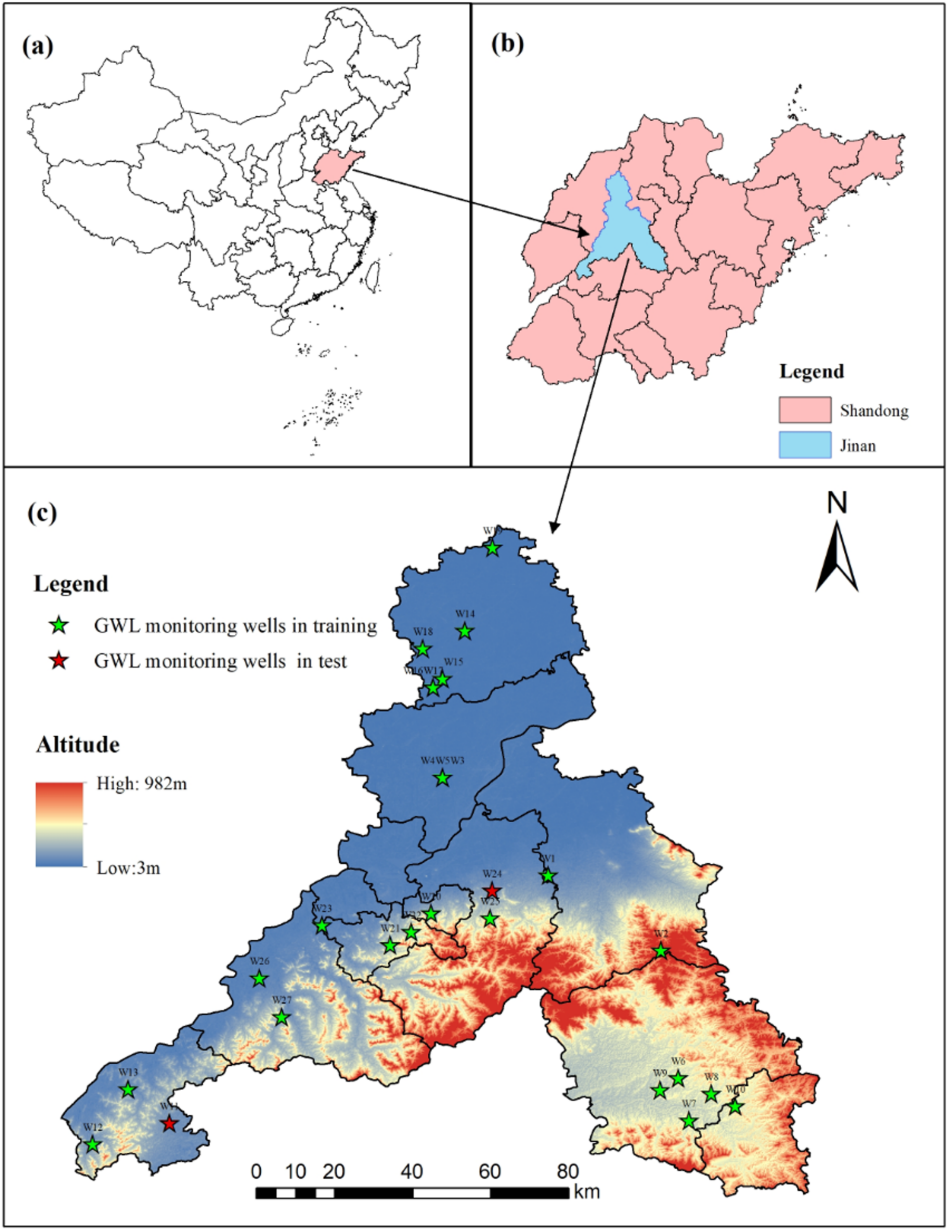
Geographical location of the study monitoring well in Jinan City, Shandong Province. (**a**) Displays the location of Shandong Province within China; (**b**) provides a zoomed-in view of Shandong Province, with a focus on Jinan, shown in the highlighted area; (**c**) presents a detailed topographic map of the study area and GWL monitoring wells, with the elevation ranging from 3 to 982 m. This figure was created using ArcGIS 10.8.1. The provincial-level administrative boundary map of China and the administrative boundary map of Shandong Province were obtained from the Resource and Environmental Science Data Platform (https://www.resdc.cn/) under a free download policy. Note: Some monitoring wells (e.g., W3, W4, W5 and W16, W17) are in very close proximity, and their markers overlap visually.

This study focused on the administrative region of Jinan City, which is located in the eastern part of North China Plain, in the middle and western part of Shandong Province, East China (Fig. 2). The geographical location ranges from 36°02’ N to 37°54’ N and from 116°21’ E to 117°59’ E, with a total area of 8,154 km^2^.The study area featured a warm-temperate continental monsoon climate situated in the mid-latitude inland area. This climatic characteristic results in significant seasonal differences in precipitation, with the majority of rainfall concentrated in the summer months (June to August), accounting for about 70% of the annual precipitation. Additionally, there is considerable inter-annual variability in precipitation. It is worth noting that atmospheric precipitation serves as the dominant recharge source for the karst aquifer system.

Jinan is situated in a transition zone between the low-mountain hills of central-southern Shandong and the alluvial plain of northwestern Shandong. The topography is higher in the south and lower in the north. The southern consists of Ordovician limestone karst aquifers, while the northern is characterized by igneous aquitards. This geological structure creates a natural hydrogeological unit with “southern recharge-northern barrier” that drives regional groundwater flow along a predominant south-to-north gradient^44^. The karst water in the central piedmont plain serves as the main water supply for Jinan, with an average GWL of 45.68 m. In contrast, the southern hilly areas are primarily composed of fracture water, with an average GWL of 227.36 m. Both karst and fracture water levels exhibit significant fluctuations.

#### Datasets

In this study, we utilized GWL observation data as the target variable and integrated multiple input variables to construct the dataset for predictive modeling. We collected static attribute data from 27 monitoring wells located within the study area (Fig. 2c), including geographical coordinates (longitude and latitude), wellhead elevation, and aquifer type. Concurrently, we obtained GWL time series recorded at 7-day intervals from January 2018 to October 2023, providing absolute elevation values (meters) relative to the national vertical datum.

To capture the complex dynamics of groundwater fluctuations, this study comprehensively considered the lag effect and driving mechanism, and incorporated three key driving factors: (1) Historical GWL data (τ time-lagged terms): characterizing temporal autocorrelation in groundwater systems; (2) Meteorological variables (precipitation, temperature, evapotranspiration): representing external climatic forcing; (3) Spatial factors (water levels of adjacent monitoring wells): quantifying the spatial correlation. Among them, the lag effect was captured through historical GWL time-lags, while the external driving mechanisms were represented by meteorological and spatial factors. These meteorological data were sourced from the National Earth System Science Data Center (http://www.geodata.cn/main/), which provides a 1 km resolution monthly dataset for the Chinese region (1901–2023), with each product containing 12 monthly bands. Data from 2018 to 2023 were selected to ensure temporal consistency with the GWL observation data. Through data preprocessing methods, the gridded meteorological data were precisely matched with the locations of each monitoring well, constructing a spatiotemporally consistent multivariate analysis dataset.

### Data preprocessing

**Fig. 3:**
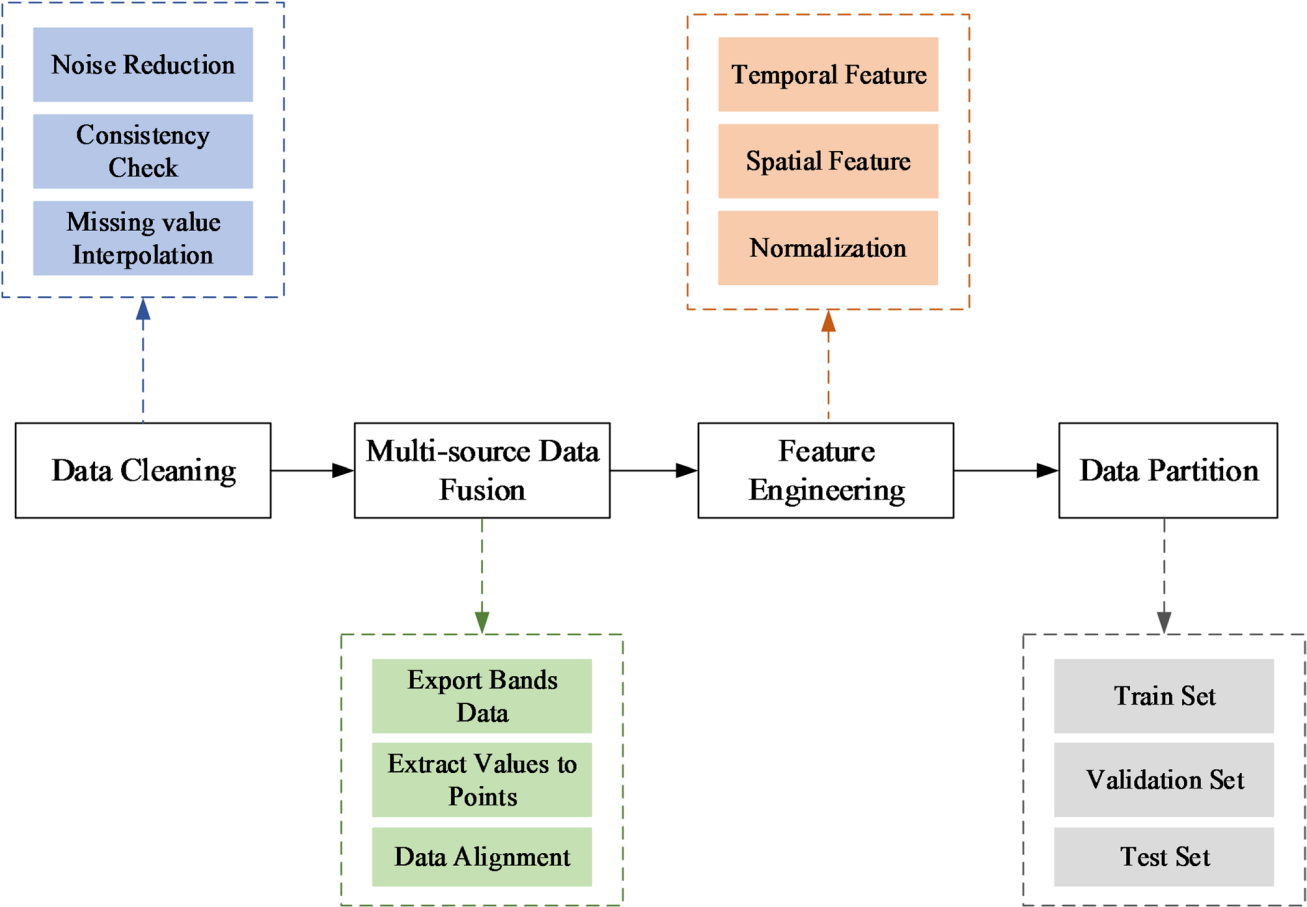
The workflow diagram of data preprocessing for GWL prediction modeling.

Data preprocessing constitutes a critical and indispensable step in the construction of deep learning models, playing a decisive role in enhancing model performance^34^. Our systematic preprocessing pipeline comprised four key phases: (1) data cleaning; (2) multi-source data fusion; (3) feature engineering; (4) dataset partitioning. The workflow of data processing is illustrated in Fig. 3.

#### Data cleaning

Given the susceptibility of groundwater level (GWL) observations to sensor errors and environmental disturbances, this study implemented rigorous noise reduction protocols to enhance signal-to-noise ratios. To address heterogeneity of multi-source data, we systematically unified the sampling frequency and measurement units across all monitoring wells, thereby eliminating potential biases from data inconsistencies^23^. Specifically: (1) depth-to-water measurements were converted to elevation head values using wellhead benchmarks, (2) high-frequency daily data were resampled to 7-day resolution using arithmetic averaging to maintain temporal consistency, and (3) a stringent quality control filter was applied to select wells with a missing rate of less than 30% and no gaps exceeding one consecutive month during 2018–2023. Missing values were then imputed using seasonal-trend decomposition (STL) to preserve the statistical properties of hydrological time series^45^.

#### Multi-source data fusion

This study employed a systematic data fusion approach to achieve spatio-temporal synchronization between meteorological variables and GWL observations. Utilizing the ArcGIS 10.8 platform, we first extracted monthly bands (2018–2023) from raster datasets for each meteorological element (precipitation, temperature, and potential evapotranspiration) through raster processing. The “Extract Values to Points” spatial analyst tool was then applied to derive precise time-series of meteorological elements at all 27 monitoring well locations. Temporal alignment was rigorously enforced by establishing unified timestamp indices that synchronize GWL records with corresponding meteorological measurements, ultimately generating a spatiotemporally coherent multivariate dataset. This fusion process ensured rigorous spatiotemporal alignment of multi-source datasets, establishing a robust foundation for subsequent spatiotemporal modeling.

#### Feature engineering

**Temporal feature engineering**: Beyond fundamental meteorological variables (precipitation, temperature, and evapotranspiration), we leveraged the time lag of GWLs to generate lagged GWL features. Autocorrelation function (ACF) analysis of GWL time series across monitoring wells revealed that 20 wells had a significant lag step of 2, while the remaining 7 wells demonstrated a significant lag step of 3. To optimize the trade-off between model complexity and feature representation capacity, this study adopted the maximal consensus lag order (lag 2) across all monitoring wells. Consequently, we constructed the GWL lag features for 7-day lagged values (GWL_lag1) and 14-day lagged values (GWL_lag2) as model inputs. Table 1 presents comprehensive statistics (Mean, Min, Max, STDEV, etc.) for both input and target variables (2018–2023), providing quantitative characterization of aquifer system dynamics and data basis for prediction model training.

**Table 1:** Statistical description of the input and output variable.

	Input variables					Output variable
	Precipitation (mm)	Temperature (ºC)	Evaporation (mm)	GWL_lag1 (m)	GWL_lag2 (m)	GWL (m)
Max	384	29	199.5	434.51	434.51	434.51
Min	0	-4	23.1	−42.89	−42.89	−42.89
Mean	63	14	98.62	76.58	76.27	76.85
Median	30	15	94.6	45.26	44.59	45.44
STD	80.44	10.23	55.36	93.02	93.00	93.03
Skew	1.87	−0.20	0.14	1.73	1.74	1.71

**Spatial feature engineering**: The spatial dataset delineated the geographical coordinates (latitude and longitude) and static attributes (elevation referenced to national geodetic datum, aquifer type classification) for all monitoring wells. Subsequently, we can precisely compute the hydraulic connectivity metrics between each monitoring well based on Euclidean distances. Table 2 provides representative examples of spatial dataset for some monitoring wells.

**Normalization**: Given the well-documented sensitivity of deep neural networks to input feature scales, this study implemented rigorous normalization using Scikit-learn machine learning library in the Python environment. This preprocessing step effectively eliminated the dimensional differences between features, ensuring the stability and convergence efficiency of model training and laying the data foundation for subsequent modeling.

**Table 2:** Representative examples of Geospatial characteristics and static attributes of monitoring wells.

	ID	Lon	Lat	Elev	AquiferType
W1	370,114,220,003	117.345	36.731	55.64	1
W2	370,114,220,004	117.606	36.558	481.13	3
…	…	…	…	…	…
W27	370,113,220,002	116.730	36.404	92.67	3

#### Dataset partitioning

To ensure systematic and reliable evaluation of the model, this study adopted the following data partitioning strategy: Initially, the time-series data of two monitoring points randomly selected from the 27 monitoring points were reserved as an independent unseen-well test set to evaluate model performance on unseen monitoring wells. The data of the remaining 25 wells were divided according to the time series, with the data of 2023 year serving as the conventional test set for final validation of prediction accuracy. Data from 2018 to 2022 were used as the model development set, which was strictly divided into a training set (80%) and a validation set (20%) in chronological order. Here, the training set facilitated the learning and optimization of model parameters, while the validation set was used to monitor the generalization ability in real time during the training process and to prevent over-fitting. After partitioning, the training, validation, conventional test, and unseen-well test sets contained approximately 5,220, 1,305, 1, 075, and 608 samples, respectively.

Ultimately, model performance was assessed through dual evaluation levels: The conventional test set was used to evaluate temporal extrapolation capability, that is, the predictive accuracy for future time points on known monitoring wells; The unseen-well test set was used to assess spatial extrapolation performance, that is, the predictive adaptability to new monitoring wells. This dual testing strategy evaluated the model performance across both temporal and spatial dimensions, ensuring the comprehensiveness and reliability of the model evaluation.

### Model construction

**Fig. 4:**
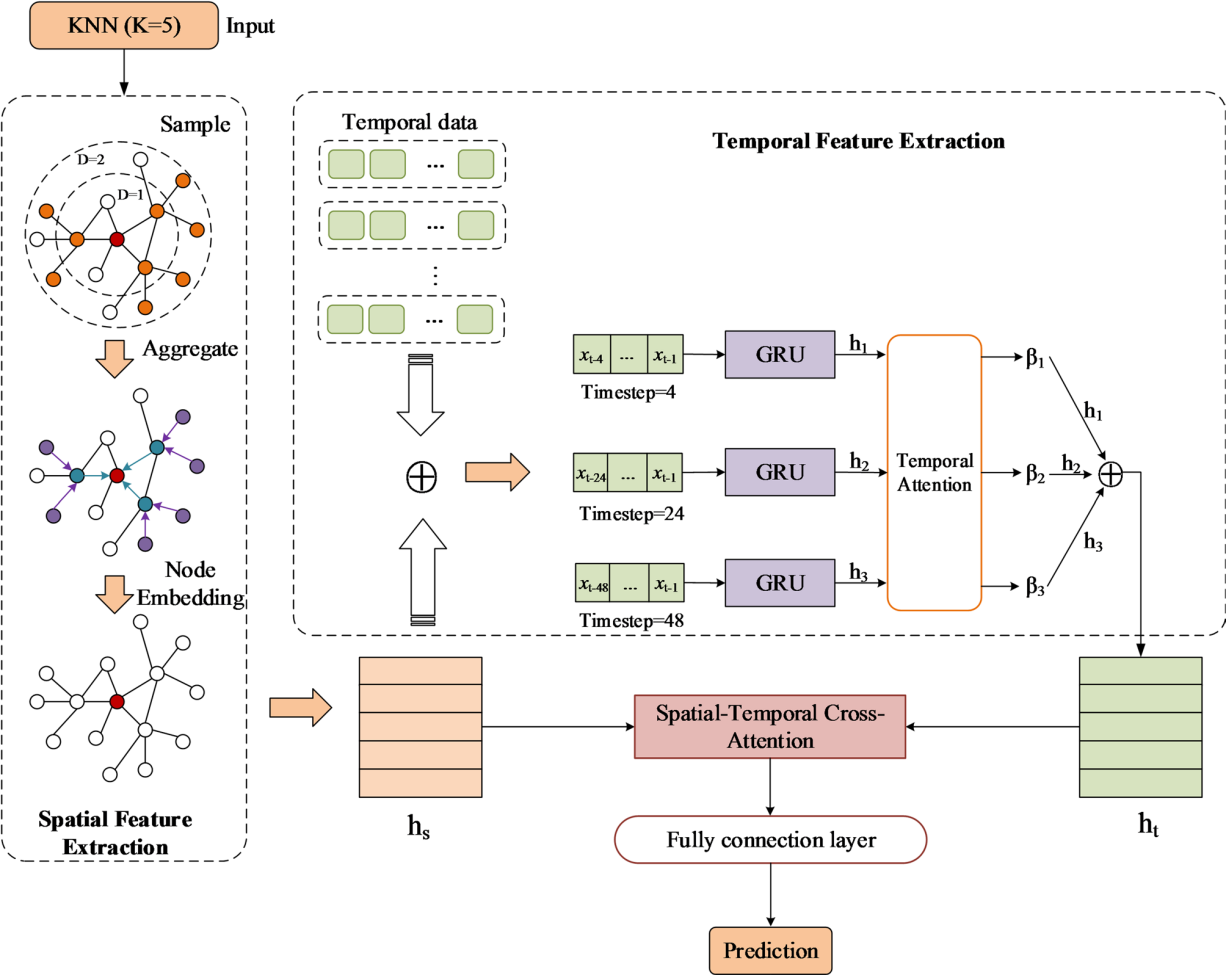
Structure of the overall model.

Inherently, GWL prediction is a complex systems problem with significant spatiotemporal coupling characteristics, where dynamic variations are simultaneously influenced by temporal evolution and spatial interactions. In the temporal dimension, GWL exhibits sequential dependence through continuous evolution, with new observations dynamically correlated to their historical states. Spatially, fluctuations in GWL at adjacent monitoring wells show significant hydraulic interdependencies. In response to these characteristics, this study designed a hybrid GWL prediction model integrating spatio-temporal features (STGPM), whose core architecture was organically composed of three key modules: spatial feature extraction, multi-scale temporal feature extraction, and spatio-temporal feature fusion. The structure of the overall model was shown as Fig. 4, which fully incorporated the spatio-temporal coupling mechanisms of the groundwater system, providing a scientifically rigorous modeling paradigm for accurate GWL prediction.

#### Construction of the K-nearest neighbor graph

To effectively capture hydraulic connectivity between monitoring wells, this study constructed a K-nearest neighbor (KNN) graph based on the geographical coordinates of monitoring wells, where each monitoring well was regarded as a node of the undirected graph. This graph structure could effectively capture the local spatial correlation among monitoring wells, providing neighborhood information for subsequent spatial feature aggregation. The specific steps were as follows:

**Coordinate extraction**: Extracted latitude and longitude coordinates of each monitoring well from their spatial information to form an N$\:\times\:$2 coordinate matrix (where N is the number of monitoring points, *N* = 25).

**K-Nearest neighbors calculation**: Utilized the nearest neighbor algorithm to calculate the K nearest neighbors for each monitoring well and obtained the Euclidean distances to these nearest neighbors.

**Edge construction**: Traversed each node and established undirected edges between it and its nearest neighbors, with edge weights set as the inverse of Euclidean distance. This weighting scheme ensured stronger connections between geographically closer nodes, thereby more accurately reflecting the spatial relationships between wells.

The final undirected graph $\:\mathcal{G}\left(\mathcal{V},\mathcal{E}\right)$ completely characterized the spatial topology structure of the monitoring well network, where $\:\mathcal{V}=\left\{{v}_{1},{v}_{2},\dots\:,{v}_{m}\right\}$ represented the monitoring wells and $\:\mathcal{E}=\left\{{e}_{\mathrm{1,2}},{e}_{i,j},\dots\:,{v}_{m,n}\right\}$ described the strength of spatial connections between them. This undirected graph served as input to the GraphSAGE model, providing accurate neighborhood information for subsequent spatial feature extraction.

#### Spatial feature extraction

This study utilized the GraphSAGE model to learn spatial feature representations of monitoring wells. The model effectively captured spatial dependencies between nodes by leveraging both the feature and structural information of nodes through neighbor sampling and feature aggregation mechanisms.

**Node sampling**: For each target node $\:\nu\:\in\:\mathcal{V}$, we employed a hierarchical sampling strategy to determine its multi-hop neighbor set $\:\mathcal{N}\left(v\right)$. The sampling process primarily focused on two parameters: the number of sampling layers $\:\mathcal{D}$ and the sampling size per layer. $\:\mathcal{D}$ represented the maximum hop count for neighbor aggregation. Experimental results demonstrated that the model achieved optimal performance when $\:\mathcal{D}=2$.

**Node aggregation**: The GraphSAGE model provided three aggregation functions: mean aggregation, LSTM aggregation, and pooling aggregation. Comparative experiments indicated that while both LSTM aggregation and pooling aggregation delivered good performance, the former exhibited significant computational inefficiency. Therefore, this study selected the pooling aggregation function, which operated by first applying a nonlinear transformation to the embedding of each neighbor node via a fully connected network, followed by the integration of neighborhood information to generate the target node embedding using max or mean pooling operations. The mathematical formulation was as follows:

1 $$\:{\mathrm{AGGREGATE}}_{d}^{pool}=max\left(\left\{\sigma\:\left({\mathrm{W}}_{pool}{h}_{{u}_{i}}^{d}+b\right),\forall\:{u}_{i}\in\:\mathcal{N}\left(\nu\:\right)\right\}\right).$$

Building upon these two processes, we first initialized the feature vector representation $\:{h}_{v}$ for each node. For each node $\:\nu\:\in\:\mathcal{V}$, its neighbor nodes $\:\mathcal{N}\left(v\right)$ were obtained through node sampling. Subsequently, the aggregation function (Eq. 1) was employed to integrate feature information from neighboring nodes. Finally, the aggregated neighborhood features were combined with the node’s own features through a nonlinear transformation to generate the updated node embedding representation, formulated as follows:

2 $$\:{h}_{v\:}^{d}=\:\sigma\:\left({\mathrm{W}}^{d}\cdot\:\mathrm{CONCAT}\left({h}_{v\:}^{d-1},{h}_{\mathcal{N}\left(\nu\:\right)\:}^{d}\right)\right).$$

#### Multi-scale temporal feature extraction

In the process of GWL prediction, the representation ability of temporal features is a critical factor influencing model accuracy. Inspired by Chen, et al. ^43^, this study employed a multi-branch GRU architecture that processed time-series data at different temporal scales in parallel, enabling joint modeling of both short-term fluctuations and long-term trends.

Considering the hydrological response characteristics of the karst aquifer system in Jinan, we defined three distinct sliding window lengths: short-term (one month), medium-term (6 months), and long-term (12 months) windows. The short-term window focused on recent GWL fluctuations. The medium-term window covered semi-annual hydrological cycles to model seasonal variation patterns, while the long-term window was dedicated to learning interannual trends. The original time series was partitioned into multiple subsequences according to these different window lengths. For example, considering a time series $\:{T}_{\mathcal{w}}=\left\{{x}_{1},{x}_{2},\dots\:,{x}_{n}\right\}$ composed of the GWL observations from monitoring well $\:\mathcal{w}$, to predict the GWL value at time $\:\mathcal{t}$, if the sliding window was set to 3, the GWL values from the three preceding time steps were extracted, forming the input sequence $\:\left\{{x}_{\mathcal{t}-3},{x}_{\mathcal{t}-2},{x}_{\mathcal{t}-1}\right\}$. These sub-sequences from the three distinct sliding windows were fed into three separate GRU branches, with each branch specifically processing the sub-sequence in a specific temporal scale. This parallel architecture enabled comprehensive modeling of both short-term perturbations (e.g., rainfall responses) and long-term evolutionary trends (e.g., seasonal cycles) in groundwater dynamics.

To further optimize feature fusion, this study introduced an attention mechanism to adaptively integrate multi-scale features. Let $\:{h}_{1},{h}_{2},\:$and $\:{h}_{3}$ denote the output feature vectors from the short-term, medium-term, and long-term GRU branches, respectively. The importance weights $\:{\beta\:}_{i}$ of each branch were calculated through the attention mechanism. The feature fusion process based on attention weights can be expressed as:

3 $$\:{h}_{t}=\sum\limits_{i=1}^{3}{\beta}_{i}\cdot\:{h}_{i}$$

This mechanism dynamically adjusted the contribution weights of features across different temporal scales, generating more discriminative spatio-temporal feature representations. The design not only preserved scale-specific information but also enhanced predictive capability through synergistic feature interactions.

#### Spatio-temporal feature fusion

The core of spatiotemporal feature fusion lies in establishing coupled representations of spatial and temporal features. This study employed a cross-attention mechanism^46^ to integrate temporal and spatial features, enabling more comprehensive feature representation. Through the aforementioned spatial and temporal feature extraction processes, supposed we obtain two feature sequences $\:{h}_{s}$ and $\:{h}_{t}$, where $\:{h}_{s}$ was the spatial feature sequence and $\:{h}_{t}$ was the temporal feature sequence. The spatio-temporal cross-attention mechanism allowed one sequence (spatial features) to serve as Query, while the other sequence (temporal features) acted as both Key and Value. The Query, Key, and Value can be expressed as:

4 $$\:Q={h}_{s}{W}_{q},\:\:K={h}_{t}{W}_{k},\:\:V={h}_{t}{W}_{v},$$

where $\:{W}_{q}$, $\:{W}_{k}$, and $\:{W}_{v}$ represented the projection matrices for Query, Key, and Value, respectively.

The cross-attention scores between spatial nodes and temporal steps were obtained by computing the similarity between Query and Key:

5 $$\:A=softmax\left(\frac{Q{K}^{T}}{\sqrt{{d}_{k}}}\right)$$

where $\:{d}_{k}$ was the dimension of the Key, serving as a scaling factor for the dot product to prevent gradient vanishing. Each element $\:A\left(i,j\right)$ in the attention matrix quantified the dependency strength between the $\:i$-th monitoring well and the $\:j$-th timestep.

Finally, temporal features were aggregated to spatial nodes through a weighted sum:

6 $$\:\mathrm{Z}=A\cdot\:V$$

### Model optimization and evaluation

#### Experimental setup

The hardware and software environment configurations employed for model optimization and evaluation were detailed in Table 3.

**Table 3:** Experiment environment.

CPU	Intel Core 11th (8 core), i9-11900, 2.50 GHz
GPU	NVIDIA GeForce GTX1660 12G
Hard disk	SSD 1 TB M.2 SATA
RAM	16G 2400 MHz DDR4 (2 × 8G)
Operating system	Microsoft Windows 10 Pro,64 bits
CUDA version	12.6
Programming language	Python 3.13.0
Deep learning framework	Pytorch 2.7.0

To ensure the reproducibility of our proposed STGPM model, this subsection provided a comprehensive description of the specific architectural configurations used for each component. The final architecture was summarized in Table 4.

**Table 4:** Detailed architecture of the STGPM.

Component	Parameter	Value
GraphSAGE	Number of Layers	2
	Embedding Dimension	64
	Aggregation Function	Max-pooling
Multi-branch GRU	Number of GRU Layers	1
	Hidden State Dimension (Short-term)	32
	Hidden State Dimension (Medium-term)	64
	Hidden State Dimension (Long-term)	64
	Dropout Rate	0.1
Attention Mechanism	Attention Dimension	128
	Attention Heads	4
Fully-Connected	Number of Layers	2
	Hidden Layer Dimension	64
	Output Dimension	1
	Activation Function (Hidden)	ReLU
	Activation Function (Output)	Linear

#### Hyperparameter optimization

To identify the optimal hyperparameter configuration for STGPM, a systematic grid search strategy was employed. This exhaustive method was selected due to the discrete and limited nature of the hyperparameter space, ensuring a comprehensive evaluation of all possible combinations to achieve globally optimal performance within the defined search domain, rather than settling for a computationally efficient but potentially local optimum.

Specifically, the grid search examined three critical parameters: learning rate, batch size, and the number of sampled neighbor nodes. The learning rate varied within the range of $\:{10}^{-4}$ to $\:{10}^{-2}$, the batch size was tested at values of [16, 32, 64, 128] to balance computational efficiency and training stability, and generalization performance. The number of samples per hop was set to 1, 3, and 5 to determine the optimal amount of neighborhood information to aggregate for spatial feature extraction. The optimization objective was trained to minimize the Mean Squared Error (MSE) between its predictions and the truth groundwater level values. Each parameter combination underwent 100 training evaluations with early stopping patience to prevent overfitting. The training and validation loss curves were meticulously monitored to ensure convergence and assess generalization performance. This optimal configuration was subsequently used to train the final model on the combined training and validation sets for all subsequent performance evaluations reported in this study.

Similarly, we adopted a rigorous approach where each model underwent an independent hyperparameter optimization process using the same grid search strategy for each baseline model. The search space for each model included key architectural parameters: the number of layers [1, 2] and the number of hidden units [32, 64, 128]. Final optimized hyperparameter configurations for all compared models were shown in Table 5.

**Table 5:** Final optimized hyperparameter configurations for all compared models.

Hyperparameter	LSTM	GRU	STGCN	STGPM
Number of layers	2	2	2 (GCN)/1 (Temporal)	2 (GraphSAGE)/1 (GRU per branch)
Hidden dimension	128	128	64 (GCN)/128 (Temporal)	64 (Spatial)/32,64,64 (Temporal)
Temporal Window	12 (steps)	12 (steps)	12 (steps)	4, 24, 48 (steps)
Learning rate	0.001	0.001	0.001	0.001
Batch size	64	64	64	64
Optimizer	AdamW	AdamW	AdamW	AdamW
Dropout rate	0.2	0.2	0.1	0.1
Early stopping patience	15	15	15	15

#### Evaluation metrics

To comprehensively evaluate model performance, this study employed a multi-dimensional metric system for quantitative analysis. The evaluation framework included the Mean Absolute Error (MAE), Root Mean Square Error (RMSE), and Coefficient of Determination (R^2^). Each metric provided distinct insights: MAE measured the absolute deviation between predicted and observed values, RMSE quantified the dispersion degree of prediction errors, and R^2^ assessed goodness-of-fit. The combination of these three indicators can objectively assess the prediction accuracy and model stability from different perspectives, providing a reliable quantitative basis for model comparison.

The MAE is the average absolute difference between predicted and observed values, quantifying the absolute magnitude of prediction errors. As the most intuitive metric, MAE is less sensitive to outliers due to the use of absolute values. Its formulation is given by:

7 $$\:\mathrm{M}\mathrm{A}\mathrm{E}=\frac{1}{n}\sum\:_{i=1}^{n}\left|{y}_{i}-\widehat{{y}_{i}}\right|,$$

where $\:{y}_{i}$ and $\:\widehat{{y}_{i}}\:$denote observed and predicted values, respectively, and $\:n$ is the sample size.

The RMSE, calculated as the square root of the mean squared errors, provides greater sensitivity to prediction variability and extreme errors. The calculation method for RMSE is as follows:

8 $$\:\mathrm{R}\mathrm{M}\mathrm{S}\mathrm{E}=\sqrt{\frac{1}{n}\sum\:_{i=1}^{n}{\left({y}_{i}-\widehat{{y}_{i}}\right)}^{2}}.$$

R^2^ quantifies the proportion of variability in the target variable explained by the model from a statistical perspective, serving as an important indicator of goodness-of-fit. An R^2^ value closer to 1 indicates a better fit, while an R^2^ close to 0 or negative suggests a poor model fit. The formula for calculating R^2^ is as follows:

9 $$\:{\mathrm{R}}^{2}=1-\frac{{\sum\:}_{i=1}^{n}{\left({y}_{i}-\widehat{{y}_{i}}\right)}^{2}}{{\sum\:}_{i=1}^{n}{\left({y}_{i}-\stackrel{-}{y}\right)}^{2}},$$

where $\:\stackrel{-}{y}$ is the mean of the observed values.

It is important to note that for model evaluation, the predictions were inverse-transformed back to the original scale (meters) before calculating the MAE, RMSE, and R^2^ metrics to ensure their physical interpretability.

#### Model interpretability

Despite the superior predictive performance of machine learning and deep learning models in groundwater level prediction, their inherent “black box” nature limits the interpretability of the model decision-making process. Model interpretability aims to uncover the underlying mechanisms between input features (such as rainfall, evaporation, and groundwater extraction) and prediction outcomes, providing a scientific basis for water resource management decisions.

This study employed the SHapley Additive exPlanations (SHAP) framework, rooted in cooperative game theory, to quantify feature contributions to the model’s prediction results by calculating the SHAP values of each feature. The advantage of this approach was that SHAP values can simultaneously reveal both the polarity (positive/negative influence) and relative importance of each feature’s impact on predictions. This method transformed opaque model behavior into interpretable, physically consistent logic, thereby comprehensively assessing model behavior.

## Results and discussion

### Analysis of precipitation, temperature, evapotranspiration, and GWL distribution

**Fig. 5:**
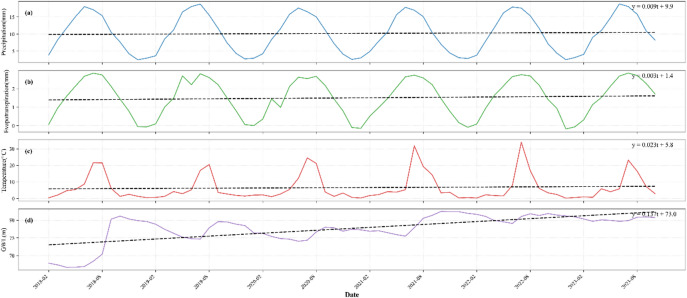
Time series of monthly average precipitation, temperature, evapotranspiration, and GWL from 2018 to 2023.

The multi-source dataset in this study included four key hydrological variables: precipitation, temperature, evapotranspiration, and GWL. As shown in Fig. 5, linear trend (LT) analysis was applied to decompose and visualize the temporal trends of these variables from 2018 to 2023, revealing the dynamic characteristics of each variable. The results indicate that both precipitation and potential evapotranspiration exhibit significant seasonal cyclical variations and are synchronous, with higher values in summer months (June-August) and lower values in winter months (December-February). This pattern aligns closely with the study area’s typical monsoon climate. Similarly, temperature data displays marked annual cyclical fluctuations, peaking in the summer and reaching their lowest points in winter. GWL shows an overall upward trend during the observation period, superimposed with periodic fluctuations that are coupled with the seasonal pattern of precipitation: water levels rise during the wet season (summer) and decline during the dry season (winter). These observations confirm that seasonal variations of meteorological factors are key drivers of GWL fluctuations, providing crucial insights into the dynamic response mechanisms of the groundwater system in the study area.

### Analysis of the model performance

#### Model training and error variation

**Fig. 6:**
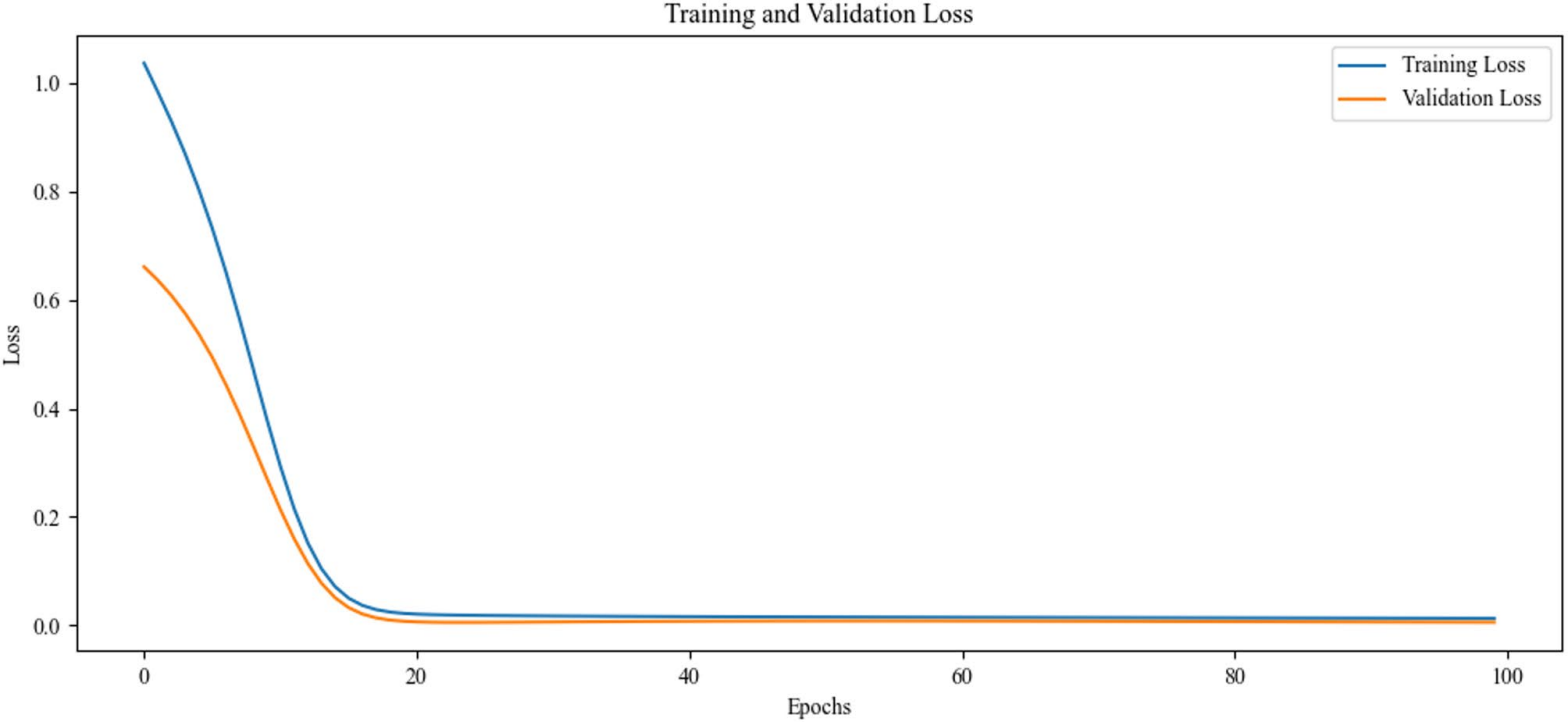
Training and validation error curves.

This study employed systematic grid search^4^ to optimize key hyperparameters of the STGPM model (as detailed in section “Hyperparameter optimization”), which indicated that the model achieves optimal performance with a learning rate of 0.001, batch size of 64, and three samples per hop. As shown in Fig. 6, the model exhibits a rapid error reduction during the initial phases of training, followed by convergence to a stable low-loss region. This training dynamic not only confirms the rationality of the parameter configuration but also highlights the model’s excellent generalization capability.

#### Performance comparison between STGPM and benchmarks

We conducted a systematic performance evaluation of STGPM by comparing it with three representative baseline models: the classical LSTM, GRU, and spatio-temporal graph convolutional network (STGCN). This comparative experiment aimed to validate the advantages of the STGPM model over existing mainstream methods in the task of GWL prediction. To ensure the fairness and comparability, all models adopted unified data partitioning strategies following the method in section “Dataset partitioning”, the data from 2023 were used as the test set, and the remaining data were divided into training and validation sets in an 8:2 ratio. Quantitative comparison was conducted using the evaluation metrics (MAE, RMSE, and R^2^) defined in section “Evaluation metrics”. In terms of model training, a completely consistent hyperparameter setting was adopted: a learning rate of 0.001, 100 training epochs, a batch size of 64, with AdamW optimizer for parameter optimization and mean squared error (MSE) as the loss function. We did not employ identical network structures across models, as their fundamental operating principles differ (e.g., sequential processing vs. graph convolution). Instead, we adopted a rigorous approach where each model underwent an independent hyperparameter optimization process using the same grid search strategy. The final reported performance for each baseline model corresponds to its individually optimal configuration (Table 5) identified through this process. This strategy ensured that we were comparing the best possible performance of each model architecture on our dataset, thereby attributing performance differences to the inherent efficacy of the models’ inductive biases for spatio-temporal groundwater level prediction, rather than to arbitrary or suboptimal structural choices. To statistically validate the performance stability and robustness of the compared models, we conducted 10 independent training runs for each model with different random seeds.

**Table 6:** Performance evaluation of different models.

Model	Stage	MAE	RMSE	*R* ^2^
LSTM	Training	0.188 ± 0.012	0.164 ± 0.015	0.889 ± 0.011
	Testing	0.154 ± 0.011	0.139 ± 0.013	0.891 ± 0.009
GRU	Training	0.161 ± 0.009	0.151 ± 0.010	0.912 ± 0.012
	Testing	0.139 ± 0.008	0.121 ± 0.009	0.928 ± 0.009
STGCN	Training	0.095 ± 0.006	0.125 ± 0.008	0.956 ± 0.008
	Testing	0.095 ± 0.007	0.103 ± 0.006	0.965 ± 0.008
STGPM	Training	**0.062 ± 0.004**	**0.064 ± 0.003**	**0.980 ± 0.001**
	Testing	**0.039 ± 0.002**	**0.052 ± 0.002**	**0.988 ± 0.001**

Table 6 presents the evaluation results of four prediction models on the groundwater dataset. The experimental results demonstrate that STGPM achieves superior predictive performance, with the lowest errors on the test set (MAE = 0.039, RMSE = 0.052) and the highest R^2^ (0.988), significantly outperforming the other benchmark models. Although STGCN shows slightly lower accuracy than STGPM, it still markedly exceeds traditional LSTM and GRU models. These findings confirm the critical role of spatial feature modeling in GWL prediction. Both STGPM and STGCN effectively capture spatial interactions between monitoring wells through graph neural networks, whereas traditional LSTM/GRU models, which rely solely on time series modeling, fail to represent this spatial dependencies, thereby resulting in limitations in predictive performance.

**Fig. 7:**
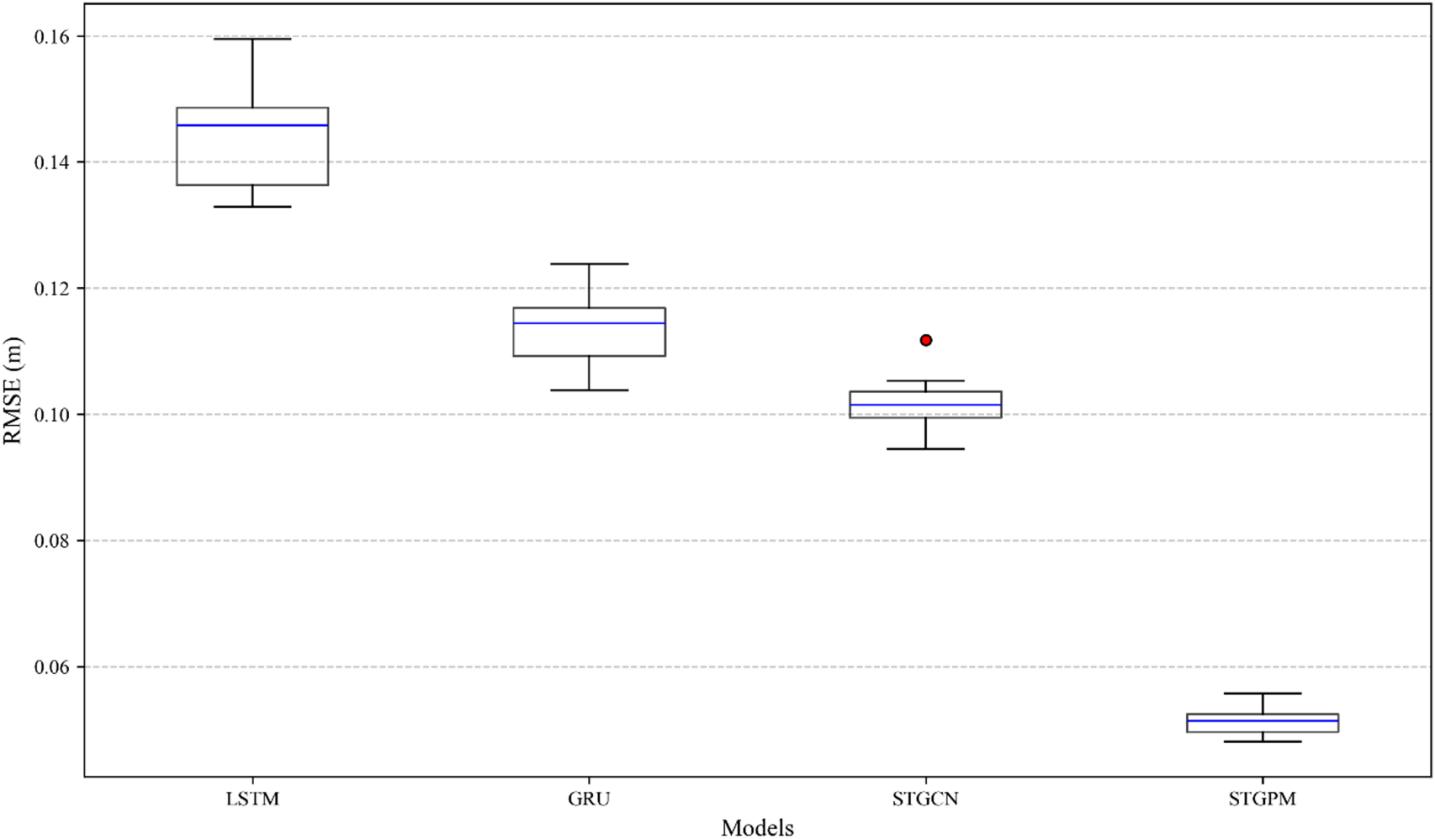
The boxplot figure of the distribution of RMSE values on the conventional test set.

Figure 7 presents the distribution of the RMSE on the conventional test set across 10 independent runs using box plots. The results clearly indicate that the proposed STGPM model not only achieved the lowest median RMSE but also exhibited the most stable performance, as evidenced by its compact box and short whiskers. This signifies that the STGPM’s superior performance is highly consistent and less sensitive to random initialization. In contrast, while the STGCN also shows relatively stable performance, its error distribution is significantly higher than that of the STGPM. The traditional LSTM and GRU models display both higher median errors and considerably larger variances. This statistical evidence reinforce the conclusion that the STGPM provides a more accurate and reliable solution for groundwater level prediction.

#### Predicted performance of new monitoring wells

The aforementioned experimental results demonstrate that existing models performed well in temporal prediction for trained monitoring wells. However, their capabilities for spatial extrapolation still need to be verified. To systematically evaluate the spatial generalization ability of the STGPM model, this study designed a dedicated prediction experiment using data from untrained monitoring wells.

Following the data partitioning scheme in Sect. 2.2.4, we randomly selected two monitoring wells to construct an unseen-well test set, with their data entirely excluded from model training. After the model was trained, the time-series data of these unseen monitoring wells were fed into the trained model for prediction. This experimental design enabled the assessment of the model’s predictive ability for GWL at entirely new spatial locations, as the model must rely on the universal patterns it has learned rather than the memory of specific monitoring wells to make inferences.

**Fig. 8:**
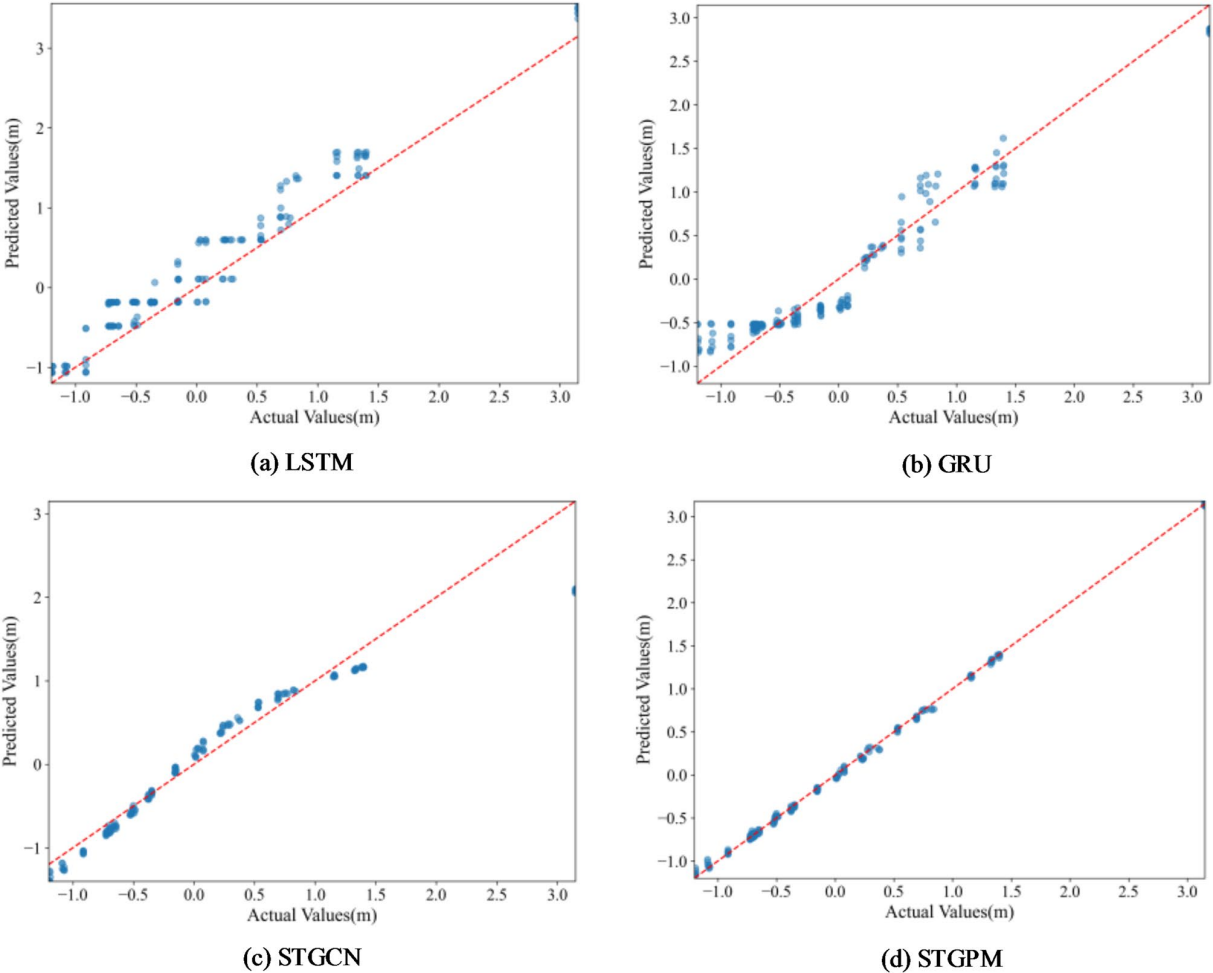
Scatter plot comparison of predicted and actual values for new monitoring wells using different models: (**a**) LSTM; (**b**) GRU; (**c**) STGCN; (**d**) STGPM.

Table 7 presents the average results over 10 independent runs. The scatter plot derived from the optimal result is shown in Fig. 8. From the results, STGPM maintains excellent prediction accuracy on untrained wells, significantly outperforming the other models. This demonstrates that STGPM possesses strong spatial generalization capabilities and can effectively adapt to monitoring well data not involved in the training process. It is worth noting that the STGCN shows limited performance improvement, likely due to constraints in its ability to characterize features at unseen nodes.

**Table 7:** Performance evaluation results of predicted new monitoring wells.

Model	MAE	RMSE	*R* ^2^
LSTM	0.158 ± 0.012	0.124 ± 0.015	0.898 ± 0.008
GRU	0.141 ± 0.005	0.125 ± 0.004	0.913 ± 0.006
STGCN	0.089 ± 0.003	0.112 ± 0.004	0.961 ± 0.002
STGPM	**0.062 ± 0.002**	**0.087 ± 0.003**	**0.980 ± 0.001**

### Analysis of ablation experiments

To validate the effectiveness of the proposed methods, a series of ablation experiments were designed. Specifically, the impact of the following three modifications on model performance was evaluated: (1) Removing GraphSAGE (STGPM^− G^): Retaining only temporal feature learning from the dataset; (2) Removing the multi-branch GRU (STGPM^− M^): Using a single-branch GRU with a fixed time window; (3) Removing the spatio-temporal attention (STGPM^− A^): Employing simple feature concatenation instead. The prediction results of these three experimental configurations on the test set are presented in Table 8.

**Table 8:** Results of the ablation experiments.

Model	MAE	RMSE	*R* ^2^
STGPM	**0.0407**	**0.0535**	**0.9869**
STGPM^− G^	0.1382	0.3718	0.8658
STGPM^− M^	0.0428	0.2069	0.9538
STGPM^− A^	0.0412	0.2021	0.9555

The experimental results demonstrate that the complete STGPM model achieves the best on the test set, significantly outperforming the variant models in the ablation studies. This indicates that STGPM can make more accurate predictions when incorporating spatial feature correlations, multi-branch GRU, and spatio-temporal attention modules. Specifically, removing GraphSAGE (STGPM^− G^) causes the most significant performance drop (MAE: 0.1382, RMSE: 0.3718, R^2^: 0.8658) compared to STGPM. This highlights the critical importance of spatial feature modeling, as GraphSAGE effectively captures spatial dependencies in the data. Without it, the model relies solely on temporal features and cannot fully utilize spatial information, leading to a substantial decrease in prediction accuracy. When the multi-branch GRU is removed (STGPM^− M^), the performance decline is relatively smaller, but still inferior to the complete model. This suggests that the multi-branch GRU enhance temporal modeling capability by extracting features at different time steps, while the single-branch GRU fails to adequately capture multi-scale temporal patterns. Removing the spatio-temporal attention (STGPM^− A^) also reduces performance, particularly in terms of RMSE and R^2^. This highlights the attention mechanism plays a crucial role in feature fusion, as simple concatenation cannot adequately capture the interactions between spatial and temporal features, whereas attention dynamically weighted feature contributions to improve performance. These ablation experiments show that GraphSAGE, multi-branch GRU, and the spatio-temporal attention mechanism all contribute significantly to STGPM’s performance. Their combined effect enable STGPM to achieve high accuracy and stability in prediction tasks.

### Analysis of feature importance and correlation

**Fig. 9:**
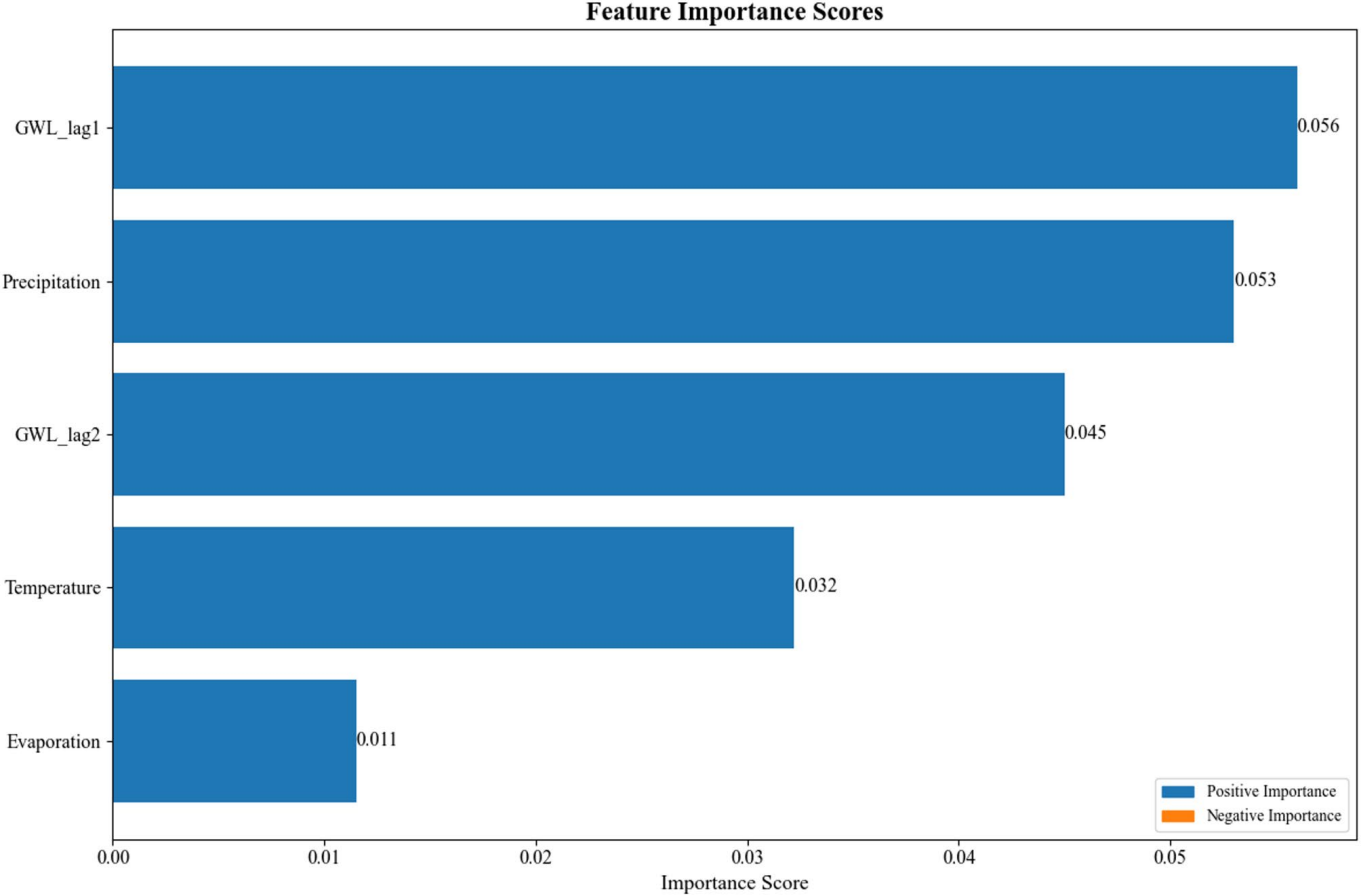
The feature importance scores of input parameters in the STGPM.

Following the construction and training of STGPM, we employed SHAP analysis to quantitatively evaluate the predictive contributions of input features, as shown in Fig. 9. The results indicate that among the five input features (precipitation, temperature, evapotranspiration, GWL_lag1, and GWL_lag2), the SHAP values of the previous water level (GWL_lag1) and precipitation are relatively high, suggesting that they are the primary driving factors affecting water level variations. Notably, GWL_lag1 has the highest SHAP value, reflecting the strong autocorrelation characteristics of GWLs. While GWL_lag2, temperature, and evapotranspiration all contribute to GWL prediction, their impacts are comparatively smaller. It is worth mentioning that while evapotranspiration generally tends to lower the water level, it may exhibit local positive correlation during the irrigation season due to artificial recharge.

**Fig. 10:**
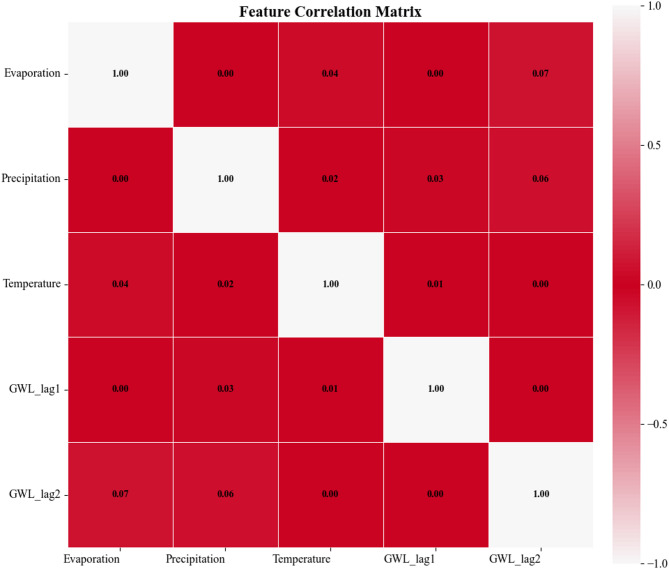
The feature correlation estimates of input parameters in the STGPM.

The presence of correlated features among input variables can compromise model stability and increase sensitivity to uncertainties. To evaluate input stability, this study further quantified linear dependencies among features using the Pearson correlation coefficient. Figure 10 displays the correlation matrix among input features, where the size of each pixel reflects similarity between features. The higher the Pearson index, the stronger their correlation. Results demonstrate the correlation coefficients for all feature pairs are below 0.1, confirming no significant correlations among the input features. The input dataset thus meet the basic requirement of feature independence for machine learning models, which effectively avoids the risk of overfitting due to feature redundancy and ensures the reliability of the prediction results.

## Conclusion

This study proposed a novel deep learning approach that significantly advanced groundwater level forecasting capabilities. By integrating GraphSAGE’s spatial representation power with a multi-branch GRU architecture featuring attention mechanisms, our framework successfully captured both the hydrological connectivity between monitoring wells and multi-scale temporal dynamics of groundwater systems. The model’s exceptional performance demonstrated the critical importance of explicitly incorporating spatial dependencies for accurate and generalizable predictions.

Through comprehensive ablation studies and benchmarking against state-of-the-art models, we established three key contributions to the field: First, the STGPM provided a novel architectural paradigm for spatio-temporal modeling in hydrogeology that effectively addressed the limitations of conventional time-series approaches. Second, through rigorous experimental validation, we quantitatively demonstrated the critical contribution of spatial features—specifically hydraulic connectivity between adjacent monitoring wells—to both prediction accuracy and model generalizability. Third, beyond providing a high-performance forecasting tool for groundwater dynamics, our methodology offered a valuable reference framework for addressing prediction challenges in other environmentally complex systems characterized by strong spatial heterogeneity, such as water quality forecasting and soil moisture prediction.

In future work, we will collect more data for model training to enhance its predictive capability and accuracy. By leveraging these improvements, researchers and decision-makers can advance their understanding and management of groundwater resources, ultimately contributing to the implementation of sustainable water management practices.

## Data Availability

The data that support the findings of this study are available from the corresponding author upon reasonable request.
